# Fine mapping of genomic regions associated with female fertility in Nellore beef cattle based on sequence variants from segregating sires

**DOI:** 10.1186/s40104-019-0403-0

**Published:** 2019-12-16

**Authors:** Gerson A. Oliveira Júnior, Daniel J. A. Santos, Aline S. M. Cesar, Solomon A. Boison, Ricardo V. Ventura, Bruno C. Perez, José F. Garcia, José Bento S. Ferraz, Dorian J. Garrick

**Affiliations:** 1 Department of Veterinary Medicine, University of São Paulo (USP), Faculty of Animal Science and Food Engineer, Pirassununga, SP Brazil; 2 Department of Animal Bioscience, Center for Genetic Improvement of Livestock, University of Guelph, Guelph, ON Canada; 3 Department of Animal and Avian Sciences, University of Maryland, College Park, Maryland, USA; 4 Department of Animal Science, University of São Paulo (USP), Piracicaba, SP Brazil; 5 Department of Sustainable Agricultural Systems, University of Natural Resources and Life Sciences, Vienna, Austria; 6 Department of Animal Nutrition and Production, School of Veterinary Medicine and Animal Science, University of São Paulo (USP), Pirassununga, Brazil; 7 Department of Support, Production and Animal Health, School of Veterinary Medicine, São Paulo State University (Unesp), Araçatuba, SP Brazil; 8 School of Agriculture, Massey University, Ruakura Ag Centre, Hamilton, New Zealand

**Keywords:** Antral follicles, Causal variants, Haplotype, Heifer pregnancy, WGS

## Abstract

**Background:**

Impaired fertility in cattle limits the efficiency of livestock production systems. Unraveling the genetic architecture of fertility traits would facilitate their improvement by selection. In this study, we characterized SNP chip haplotypes at QTL blocks then used whole-genome sequencing to fine map genomic regions associated with reproduction in a population of Nellore (*Bos indicus*) heifers.

**Methods:**

The dataset comprised of 1337 heifers genotyped using a GeneSeek® Genomic Profiler panel (74677 SNPs), representing the daughters from 78 sires. After performing marker quality control, 64800 SNPs were retained. Haplotypes carried by each sire at six previously identified QTL on BTAs 5, 14 and 18 for heifer pregnancy and BTAs 8, 11 and 22 for antral follicle count were constructed using *findhap* software. The significance of the contrasts between the effects of every two paternally-inherited haplotype alleles were used to identify sires that were heterozygous at each QTL. Whole-genome sequencing data localized to the haplotypes from six sires and 20 other ancestors were used to identify sequence variants that were concordant with the haplotype contrasts. Enrichment analyses were applied to these variants using KEGG and MeSH libraries.

**Results:**

A total of six (BTA 5), six (BTA 14) and five (BTA 18) sires were heterozygous for heifer pregnancy QTL whereas six (BTA 8), fourteen (BTA 11), and five (BTA 22) sires were heterozygous for number of antral follicles’ QTL. Due to inadequate representation of many haplotype alleles in the sequenced animals, fine mapping analysis could only be reliably performed for the QTL on BTA 5 and 14, which had 641 and 3733 concordant candidate sequence variants, respectively. The KEGG “Circadian rhythm” and “Neurotrophin signaling pathway” were significantly associated with the genes in the QTL on BTA 5 whereas 32 MeSH terms were associated with the QTL on BTA 14. Among the concordant sequence variants, 0.2% and 0.3% were classified as missense variants for BTAs 5 and 14, respectively, highlighting the genes *MTERF2*, *RTMB*, ENSBTAG00000037306 (miRNA), ENSBTAG00000040351, *PRKDC*, and *RGS20*. The potential causal mutations found in the present study were associated with biological processes such as oocyte maturation, embryo development, placenta development and response to reproductive hormones.

**Conclusions:**

The identification of heterozygous sires by positionally phasing SNP chip data and contrasting haplotype effects for previously detected QTL can be used for fine mapping to identify potential causal mutations and candidate genes. Genomic variants on genes *MTERF2*, *RTBC*, miRNA ENSBTAG00000037306, ENSBTAG00000040351, *PRKDC*, and *RGS20,* which are known to have influence on reproductive biological processes, were detected.

## Background

Fertility is a major determinant of female reproductive efficiency, particularly in Zebu (*Bos indicus*) cattle, where heifers take longer to reach puberty compared to Taurine (*Bos taurus*) animals [1, 2]. Improvements in reproductive rates can increase lifetime productivity, increase the number of animals that can be harvested for meat, reduce the number of replacement females that must be retained, and collectively increasing whole system profitability [3]. Direct assessment of fertility through comprehensive phenotypic observation is often a difficult task [4], whereas indicator traits of fertility, such as number of follicles, could allow for the collection of larger volumes of reproductive data, resulting in more reliable estimation of breeding values for fertility traits, facilitating selection [5].

Advances in genomic technologies have led to the identification of thousands of DNA markers (single nucleotide polymorphisms – SNPs) spread across the genome, which can be rapidly and inexpensively genotyped [6]. Such SNP information is commonly reported as unphased genotypes, which means that it is not immediately apparent which heterozygous allele was paternally or maternally inherited. When a process of phasing is performed with the genotype data, the unobserved haplotypes can be reconstructed. Fine mapping of haplotypes that span QTL regions can be performed to detect possible causative mutations on traits of interest [7].

Haplotype blocks (haploblock) result from the joint inheritance of nearby loci without the occurrence of recombination events within blocks. At population level, there are many possible combinations of alleles at nearby loci and therefore many potential alternative haplotypes. Haplotype alleles may exhibit major effects on animal performance, often being associated with traits of economic interest [8, 9]. Knowledge of the haplotypes straddling a QTL in half-sib families can be used to segregate offspring of parents that are heterozygous for the QTL in that specific genomic region [10].

A deeper understanding of the effects of haplotype alleles on target phenotypes can be achieved by determining the causal quantitative trait nucleotides (QTN), i.e. polymorphisms that explain the effect of a QTL. With the advent of whole-genome sequencing technology, tens of thousands of base pairs that are positional candidates for the QTL can be readily compared between individuals known to be segregating the QTL [11]. Currently only a small number of QTN that affect polygenic traits have been identified [12]. Using causative QTN rather than SNP markers has the potential to improve the accuracy of genomic selection and aid in elucidating the biological mechanism affecting variation on a trait [13–15].

In this study, we phase genotypes to reconstruct haplotypes in regions of previously reported QTL associated with heifer pregnancy (HP) or number of antral follicles (NF) in a Nellore cattle population. We contrast the two haplotype alleles carried by each sire at each QTL to determine those sires that are segregating the QTL effects and therefore segregating the causal mutation. Positional whole-genome sequence data were then used to identify those sequence variants that were concordant with the QTL segregation status.

## Material and methods

### Dataset

The dataset used for this study was previously described by Oliveira Júnior et al. [16], and consisted of HP records on 1337 Nellore heifers, with a subset of 940 of these animals also being measured for NF. Both traits were measured either using transrectal ultrasound or palpation 40 days after insemination. Heifer pregnancy was a binary trait, being analyzed using a threshold model after assigning a value of 1 (success) to heifers that were diagnosed pregnant and 0 (failure) to those with a negative (not pregnant) diagnosis. The NF was a count of all visible follicles (≥3 mm of diameter) in both ovaries on day 4 of the synchronization protocol [16]. The animals were raised in three separate herds, with the heifers being an average age of 16 months old when phenotypes were collected.

The 1337 heifers were genotyped on a GGP *Bos indicus* HD array (74677 SNPs), which is a subset of the 777962 SNPs from the Illumina® BovineHD BeadChip, chosen for being particularly informative in *Bos indicus* cattle. The heifers were the offspring from 78 sires, among which 42 of these were previously genotyped using the BovineHD BeadChip. The sire genotypes were reduced to the same filtered set of markers that passed quality control for the heifers (64800 SNPs).

The genotypes of sire-offspring pairs as recorded in the pedigree were tested for opposite homozygosity using FImpute 2.2 [17] to detect pedigree errors considering a Mendelian error rate threshold of 0.1%. The sire recorded in the pedigree was set to missing when a pedigree error was detected.

Contemporary groups (CG), that represented the group of animals that were born and managed together, were formed as the subclass combinations of birth year within herd, resulting in 12 herd-year groups. Records from any CG without phenotypic variability were eliminated from the analyses. Furthermore, animals with age exceeding 3.5 standard deviations from the overall mean age in the CG were excluded from the dataset. More details about the dataset and edits can be found in Oliveira Júnior et al. [16].

## Imputation of non-genotyped sires

Some 36 of the 78 sires with genotyped offspring were not themselves genotyped. Among those, the 19 ungenotyped sires that had more than five genotyped progeny [18, 19] were imputed to the SNP density of their offspring (64800 SNPs) using FImpute 2.2 software [17].

The accuracy of imputation was tested by similarly imputing the genotypes (64800 SNPs) of the 42 genotyped sires that had more than five progeny. The accuracy of their imputation was quantified as the correlation between observed and imputed genotypes [20].

## Genomic analyses to phase markers and estimate effects of haplotype blocks

Haploblocks considered in this paper were limited to those reported in Oliveira Júnior et al. [16]. These represented those QTL that accounted for > 1% of the total additive genetic variation for each trait (HP and NF). Phasing of SNP chip alleles to reconstruct the haplotypes was performed with half-sib pedigrees using version 3 of *findhap* [21], considering the SNP chip genomic information on both the heifers and their genotyped or imputed sires.

Haplotype mismatches were identified when supposed half sib progeny did not receive either of the paternal haplotype alleles carried by their putative sire at the given haploblock region. Observations for heifers where any haplotype allele did not match its sire, or any that were observed in less than three offspring of any particular sire, were omitted from subsequent analyses.

The effects of all the paternal haplotypes were estimated for each haploblock by fitting models with the dosage of all the haplotype alleles in that haploblock as fixed effects, using Gensel software [22]. In the same model, to account for population structure, all the panel-based SNPs except those in the haploblock being fitted were simultaneously fitted as random effects in a mixture model. The Bayes B [20] model was:

1 $$ {y}_i= Fb+ Hh+\sum \limits_{j=1}^n{x}_{ijk}{s}_j+{e}_i $$

where *y*_*i*_ is the trait record for heifer *i*; *b* is the vector that includes class effects of the contemporary groups and a regression coefficient for heifer age (months) at artificial insemination; *F* is the incidence matrix relating *b* to *y*; *h* is the vector of the fixed effects of each haplotype allele fitted, the incidence matrix *H* comprises a column for each haplotype allele representing the dosage (0, 1, 2) of the allele for each individual; *n* is the number of SNP chip loci outside the QTL region (i.e. not in the haploblock); *x*_*ijk*_ is the genotype covariate of animal *i* at SNP chip locus *j*, with genotype allele dosage indicator *k* (coded 0, 1 or 2); *s*_*j*_ is the allele substitution effect of SNP chip marker *j*, assuming ${s}_j\mid \pi, {\sigma}_j^2\sim {\delta}_jN\left(0,{\sigma}_j^2\right)$, where ${\sigma}_j^2$ is marker effect variances when *δ*_*j*_ = 1, and ${s}_j=\left(1-{\delta}_j\right)\ N\left(0,{\sigma}_{si}^2=0\right)$ when *δ*_*j*_ = 0; *δ*_*j*_ is an indicator variable for locus *j*; and *e*_*i*_ is the random residual effect for animal *i*, assuming ${e}_i\mid {\sigma}_e^2\sim N\left(0,{\sigma}_e^2\right)$. The prior for *δ*_*j*_ was:

2 $$ \left({\delta}_j|\pi \right)\Big\{{\displaystyle \begin{array}{c}1; probability\left(1-\pi \right)\\ {}0; probability\ \left(\pi \right)\ \end{array}}, where\ \pi\ was\ 0.999, as\  in\ Oliveira\ Junior, et\  al.\left[16\right] $$

Heifer pregnancy was analyzed using a threshold model, which related the observed categorical success or failure scores to an underlying continuous normal scale, whereas NF was modeled as a continuous variable. Samples from the first 2000 iterations of the Markov chain were discarded (burn-in) and every 100^th^ sample from the following 88000 samples were used for inference. The posterior distribution of the contrast between the effects of the two haplotype alleles for each sire was constructed from the Markov chain samples of each haplotype allele to identify sires that were segregating alternative QTL alleles at the haploblock, using a posteriori probability (*alpha*) < 0.10 as the threshold level.

The haplotype alleles were identified in further sections using the notation Trait_BTA_Allele, (where Trait is HP or NF; BTA is 5, 8, 11, 14, 18, or 22; Allele is 1,2,3…number of alleles).

## QTL fine mapping using whole-genome sequence data

Whole-genome sequencing (WGS) data of 26 Nellore sires was available, comprising of six bulls that were among the 42 genotyped sires included in the previous analyses, and another 20 bulls that had more distant degrees of relationships with the heifers, mainly as grandsires. The WGS data was obtained using paired-end sequencing from an Illumina HiSeq 2500 platform, following recommended manufacturer protocols (Illumina, Inc., San Diego, CA). A total of 26115176 variants were identified to be segregating in these 26 animals, including 91.6% that were SNPs and another 8.4% that were genomic insertions or deletions. The overall average depth of sequencing coverage was 14.7X.

A visual quality control (QC) of raw data was done using the FastQC package [23]. Sequence reads were aligned to the *Bos taurus* reference genome assembly (UMD 3.1) using BWA-MEM software (v0.7.15) [24]. The PCR duplicates were identified and filtered using Picard tools v2.6.0 (http://picard.sourceforge.net/). Mapped regions were analyzed in the variant calling process using GATK v3.6 Haplotype caller [25]. The identified variants were then filtered using GATK v3.6 VariantFiltration, considering the quality of called variant (QUAL) ≥ 20 and read depth (DP) ≥ 4. The remaining positional variants (after QC) were imputed into each of the haplotype alleles carried by the sires of the heifers with phenotypic fertility observations. Any particular positional sequence variant must either be present, or absent, in each of the haplotype alleles. The presence or absence of each variant in each haplotype allele was determined by linear regression. That involved constructing phenotypic vectors of length equal to the number of sequenced sires, one vector for each sequence variant, that contained the values 0, 1 or 2, representing the number of non-reference sequence alleles observed in the sequence reads of that sire. 

## Regression analysis to imputing sequence variants into known haplotypes

The presence or absence of any positional sequence variants in every sire haplotype allele at a QTL were imputed using linear regression. The dosage of the sequenced variant (i.e. 0, 1, or 2 according to the number of allele copies) at each locus in the QTL region was regressed on the dosage of the haplotype alleles at that QTL. This regression was repeated for every sequence variant that was mapped in any of the six QTL regions. For this purpose, haploblocks that spanned several Mb were partitioned into narrower fragments (~ 1 Mb) otherwise it was rare for sequenced sires to share the same haplotype alleles. These analyses were only undertaken for haplotype fragment (~ 1 Mb) that had been observed on at least three occasions. The following model was fitted separately at each sequence locus within its corresponding QTL region:

3 $$ y= Qd+e, $$

where *y* is a vector of length equal to the total number of sequenced sires and contained the dosage of the alternate allele (coded 0, 1 or 2) at a sequenced locus considered as a phenotype; *Q* is a matrix containing haplotype allele dosages (coded 0, 1 or 2) for each sequenced sire for all *N* haplotype fragments at the QTL; *d* is the vector of regression coefficients for the sequenced allele in each of the *N* haplotype alleles, which is expected to be 0 or 1 providing there were no errors in the sequencing calls, no phasing errors in the QTL haplotypes, and all copies of each haplotype allele carried the same sequence SNP; and *e* is the vector of residual imputation error. There were six different Q matrices representing the six different QTL regions. There were many vectors of phenotypes representing sequence variants for each QTL region. Regression coefficients that were exactly 0 or 1 and with standard errors < 0.0001 were used to impute the sequence SNPs to haplotype alleles whereas sequence loci with regression coefficients of intermediate values or with larger standard errors were assumed to be unreliably imputed and were therefore not considered in any further analyses.

## Imputed sequence variants concordant with segregation status of sires

As in Weller et al. [12], it was assumed that there were not multiple QTN in a single QTL. That is animals that were homozygous for a QTL were assumed to be homozygous at the QTN, and animals that were heterozygous for a QTL were assumed heterozygous at the QTN. This implies that each QTL has only one biallelic QTN, shared by all sires that were segregating that QTL. As we were interested in the difference between the significant (based on the contrast analyses) haplotype alleles, only sequence variants that were heterozygous were retained to construct the list of concordant positional sequence variants for further analysis. The absence (predicted value close to zero) or the presence (predicted value close to one) of a particular SNP allele in the haplotype was used to fine map the likely mutations responsible for the difference between alleles. Although causative mutations may result from indels, or copy number variations, only SNPs were considered [26].

## Gene search and functional enrichment

The candidate causal mutations were annotated using *Ensembl v.88* Variant Effect Predictor (*VEP*) [27] and assigned to bovine genes based on the UMD3.1 assembly [28] using the Bioconductor R package biomarRt [29, 30]. The candidate causal mutations were only assigned to a particular gene if they were located within the genomic sequence of the gene. The effect of amino acid changes were predicted for non-synonymous mutations using the SIFT score [31], a sequence homology-based algorithm that can determine whether an amino acid substitution in a protein is likely to be deleterious (scores < 0.05) or tolerated (scores ≥0.05).

Functional terms, defined as groups of genes that share a biological process or molecular function, can be used to categorize genes using over-representation analysis (ORA). We evaluated ORA considering two databases: Medical Subject Headings (MeSH) [32] and Kyoto Encyclopedia of Genes and Genomes (KEGG) [33]. The significance (*P-* value < 0.05) for reporting *g* significant genes in a certain functional term was estimated by:

4 $$ P-\mathrm{value}=1-\sum \limits_{i=0}^{g-1}\frac{\left(\begin{array}{c}S\\ {}i\end{array}\right)\left(\begin{array}{c}N-S\\ {}k-i\end{array}\right)}{\left(\begin{array}{c}N\\ {}k\end{array}\right)} $$

where *S* is the total number of target genes, *N* is the total number of genes that were analyzed, and *k* is the total number of genes in the term considered [34, 35]. The MeSH analysis was carried out using the R package *meshr* [36] while the KEGG analysis was performed using the *ppiPre* package [37]. Semantic similarities among MeSH terms were estimated using the R package *MeSHSim* [38].

## Results

Pedigree inconsistencies were detected from the presence of opposite homozygous SNP chip genotypes in about 9% of the sire-offspring pairs. All genotyped sires had genotyped offspring that were consistent with the pedigree-records, suggesting there had not been any sample mismatch errors in the sires’ genotypes. The average (±standard deviation) SNP chip imputation accuracy for the 42 genotyped sires was 0.96 (±0.03). Accordingly, a total of 19 non-genotyped sires with at least five genotyped progeny were imputed for the 64800 SNPs.

The average width for the three HP and the three NF haploblocks was 4.04 Mb and 3.91 Mb, respectively. This represented around 4.6% of the bovine chromosomes on which they were located. The six haploblocks harbored on average 98 SNPs (Table 1). The posterior distributions for the contrasts between the predicted effects of the sires’ haplotype alleles identified 15 different alleles (*alpha* < 0.10) that were associated with HP and 20 alleles that were associated with NF (Table 2).

**Table 1:** Position, size and number of SNPs of the estimated haplotype blocks for heifer pregnancy (HP) and the number of antral follicles (NF)

Trait	BTA	Position, bp	Size, Mb	#SNPs
HP	5	70,420,628 – 74,518,588	4.10	99
	14	20,344,343 – 24,418,370	4.07	97
	18	54,016,934 – 57,969,493	3.95	97
NF	8	4,159,163 – 7,663,923	3.50	99
	11	69,411,914 – 73,659,167	4.24	99
	22	11,935,606 – 15,926,677	3.99	95

BTA = chromosome number; Position = start and end position in base pair (bp); Size = QTL size in megabases (Mb); #SNPs = number of SNP markers within the QTL region.

**Table 2:** QTL alleles of heterozygous sires (*alpha* < 0.10) for heifer pregnancy (HP) and number of antral follicles (NF)

Sire	HP_BTA5	HP_BTA14	HP_BTA18	NF_BTA8	NF_BTA11	NF_BTA22
1	1 vs. 2	NS	NS	NS	49 vs. 45	NS
2	1 vs. 3	NS	NS	NS	40 vs. 45	NS
3	1 vs. 4	NS	NS	NS	NS	NS
4	5 vs. 6	NS	NS	NS	40 vs. 44	NS
5	7 vs. 8	NS	NS	NS	52 vs. 53	NS
6	9 vs. 10	NS	NS	36 vs. 37	NS	NS
7	NS	11 vs. 12	NS	NS	NS	NS
8	NS	13 vs. 14	NS	30 vs. 32	50 vs. 51	NS
9	NS	15 vs. 14	NS	NS	NS	NS
10	NS	16 vs. 17	NS	NS	NS	NS
11	NS	18 vs. 19	22 vs. 23	38 vs. 39	NS	NS
12	NS	20 vs. 21	23 vs. 24	NS	NS	NS
13	NS	NS	25 vs. 26	NS	NS	NS
14	NS	NS	27 vs. 24	NS	NS	NS
15	NS	NS	28 vs. 29	NS	48 vs. 45	NS
16	NS	NS	NS	30 vs. 31	NS	NS
17	NS	NS	NS	33 vs. 34	NS	60 vs. 61
18	NS	NS	NS	35 vs. 34	NS	NS
19	NS	NS	NS	NS	40 vs. 41	NS
20	NS	NS	NS	NS	40 vs. 42	62 vs. 63
21	NS	NS	NS	NS	40 vs. 43	NS
22	NS	NS	NS	NS	46 vs. 45	59 vs. 58
23	NS	NS	NS	NS	47 vs. 45	NS
24	NS	NS	NS	NS	54 vs. 42	NS
25	NS	NS	NS	NS	55 vs. 41	NS
26	NS	NS	NS	NS	44 vs. 41	NS
27	NS	NS	NS	NS	NS	56 vs. 57
28	NS	NS	NS	NS	NS	64 vs. 65

BTA = chromosome number; NS = non-significant.

For HP, the haplotype allele labeled *1* on BTA 5 (HP_5_1) was present in three sires (sires 1, 2, and 3). For all these sires, HP_5_1 had a favorable effect on HP when compared to those sires’ alternate haplotype alleles. Similarly, alleles HP_5_5, HP_5_7 and HP_5_9 in sires 4, 5 and 6 had favorable effects on HP in relation to their alternate alleles (Additional file 1: Figure S1).

Six sires (sires 7 to 12) had significant differences between their haplotype alleles for HP at the BTA 14 QTL (Additional file 2: Figure S2). The allele labeled *14* (HP_14_14) had a smaller effect in two different sires (sires 8 and 9), suggesting it contained an unfavorable allele for this trait.

In addition to segregating the QTL on BTA 14 for HP, sires 11 and 12 were segregating the QTL on BTA 18 (Additional file 3: Figure S3). Although the allele labeled *23* of BTA 18 (HP_18_23) had a favorable result when contrasted with HP_18_24 in sire 12, it was inferior to HP_18_22 in sire 11. The HP_18_24, in turn, had inferior values in both sires 12 and 14, where it was contrasted with HP_18_27, suggesting that it was an unfavorable allele for HP.

For NF, the contrast analyses for the BTA 8 QTL resulted in the identification of six sires (sires 8, 6, 11, 16, 17 and 18) with haplotype alleles significantly different from their corresponding alternate alleles (Additional file 4: Figure S4). Two of these, sires 8 and 11, were also segregating the HP QTL on BTA 14, while sires 6 and 17 reported segregating on BTA 5 and 22 for HP and NF, respectively. Allele *30* (NF_8_30) had positive effects on NF in sires 16 and 8, whereas allele *34* (NF_8_34) had negative effects in sires 17 and 18.

Of the 20 haplotype alleles that positively affected NF, half were on BTA 11, carried by 14 different sires (Additional file 5: Figure S5). Allele labeled *40* (NF_11_40) had a positive effect on five animals (sires 2, 4, 19, 20 and 21), suggesting the haplotype carried a favorable sequence for NF. In contrast, allele *45* (NF_11_45) showed inferior results in five different animals (sires 1, 2, 15, 22 and 23), suggesting it harbored an unfavorable allele for NF. Moreover, alleles *41* (NF_11_41) and *42* (NF_11_42) had inferior effects on the alternative alleles in three (sires 19, 25 and 26) and two (sires 20 and 24) animals, respectively. Some sires were also heterozygous for QTL associated with HP. Sires 1, 2, 4 and 5 were segregating the QTL on BTA 5, and sires 8 and 15 were segregating the QTL on BTA 14 and 18, respectively.

On BTA 22, the contrast analyses identified five alleles (NF_22_56, 59, 60, 62 and 64) positively affecting NF (Additional file 6: Figure S6). Those five alleles were present in five different animals (sires 17, 20, 22, 27 and 28), where sires 20 and 22 were also heterozygous for the QTL on BTA 11, and sire 18 for the QTL on BTA 8.

### Sequence variant-haplotype regression analysis

The average number of positional mapped reads per sample before and after filtering for the HP QTL were: 49717 and 49559 for BTA 5; 53936 and 53750 for BTA 14; 51524 and 51246 for BTA 18, respectively. The haplotype alleles identified to be segregating for NF were not well represented in the sequenced sires. Consequently, fine mapping for those NF QTL was not able to be performed.

The results of the regression analyses for the significant haplotype alleles on BTA 5 identified 641 variants (0.015% of the sequence SNP in the window) as possible causal mutations affecting HP. From these, 527 variants were in sire 1 and 468 in sire 4, with 354 common to both. Among these 354 common variants to both sires, 202 fell within genes. For the alleles on BTA 14, there were 3733 variants (0.09% of the sequence SNP in the window) that were detected, being 3432 variants from sire 7, another 3184 from sire 8 and 3709 from sire 10. The majority of the identified variants where shared between them. Some of those variants (1128) were annotated as being within genes (Table 3). None of the regression coefficients on the BTA 18 variants reached the imposed imputation threshold, and were therefore not considered to be reliable enough for concordance testing.

**Table 3:** Number of the sequenced variants (#SNPs) within genes annotated in the heifer pregnancy QTL of chromosomes (BTA) 5 and 14

Ensembl ID	Gene (#Entrez)	Position*	#SNPs
BTA 5			
ENSBTAG00000014411	*TMEM263* (539347)	70,573,524 – 70,593,134	6
ENSBTAG00000010144	*MTERF2* (782988)	70,596,429 – 70,602,003	6
ENSBTAG00000010149	*CRY1* (535947)	70,606,115 – 70,701,030	8
ENSBTAG00000037306	*–*	70,793,315 – 70,793,420	1
ENSBTAG00000027064	*BTBD11* (539986)	70,923,456 – 71,257,389	111
ENSBTAG00000004654	*PWP1* (514147)	71,280,815 – 71,300,027	1
ENSBTAG00000012999	*PRDM4* (540731)	71,324,566 – 71,347,630	1
ENSBTAG00000011070	*RTCB* (525106)	71,366,686 – 71,388,046	1
ENSBTAG00000011071	*BPIFC* (516784)	71,391,550 – 71,427,743	1
ENSBTAG00000020636	*SYN3* (100138309)	71,475,847 – 71,926,718	19
ENSBTAG00000021953	LARGE1 (506466)	72,157,229 – 72,769,395	47
BTA 14			
ENSBTAG00000040351	–	20,717,785 – 20,720,429	2
ENSBTAG00000044106	*SPIDR* (512910)	20,740,960 – 20,987,465	197
ENSBTAG00000017016	*H3F3C* (512741)	21,033,277 – 21,034,329	1
ENSBTAG00000017019	*PRKDC* (512740)	21,038,513 – 21,164,037	197
ENSBTAG00000046325	–	21,164,873 – 21,192,165	6
ENSBTAG00000023218	*UBE2V2* (286803)	21,225,303 – 21,245,522	10
ENSBTAG00000008693	*EFCAB1* (505272)	21,456,452 – 21,464,699	16
ENSBTAG00000038286	*PPDPFL* (100126183)	21,617,777 – 21,622,267	11
ENSBTAG00000002448	*SNTG1* (517353)	22,212,868 – 22,350,058	334
ENSBTAG00000017492	*PCMTD1* (521261)	22,669,363 – 22,717,576	2
ENSBTAG00000005560	*ST18* (536336)	22,815,477 – 22,872,779	10
ENSBTAG00000000878	*RB1CC1* (539858)	23,147,992 – 23,177,073	26
ENSBTAG00000000914	*OPRK1* (540519)	23,373,836 – 23,395,443	8
ENSBTAG00000003450	*ATP6V1H* (282657)	23,511,301 – 23,558,678	66
ENSBTAG00000003454	*RGS20* (614441)	23,561,303 – 23,613,020	60
ENSBTAG00000003460	*TCEA1* (505722)	23,620,858 – 23,637,730	7
ENSBTAG00000004243	*LYPLA1* (539992)	23,651,477 – 23,668,800	16
ENSBTAG00000011203	*RP1* (280916)	23,990,193 – 23,999,338	6
ENSBTAG00000047303	–	24,019,278 – 24,100,855	38
ENSBTAG00000044050	*XKR4* (517598)	24,295,567 – 24,610,955	115

#Entrez = Entrez gene ID; Position = gene start and end position in base pair; * = position based on UMD 3.1 assembly.

### Gene set analysis

The over-representation analysis, based on the MeSH database, did not detect any terms associated with the gene list from BTA 5. However, 32 significant MeSH terms were identified in the “Chemicals and Drugs” category based on the gene list of BTA 14 (Additional file 7, Table S1). Considering the KEGG database, the terms “Circadian rhythm” and “Neurotrophin signaling pathway” were significantly associated with the genes present in the gene list of BTA 5, whereas “Non-homologous end-joining” and “RNA polymerase” were present for BTA 14.

**Fig. 1:**
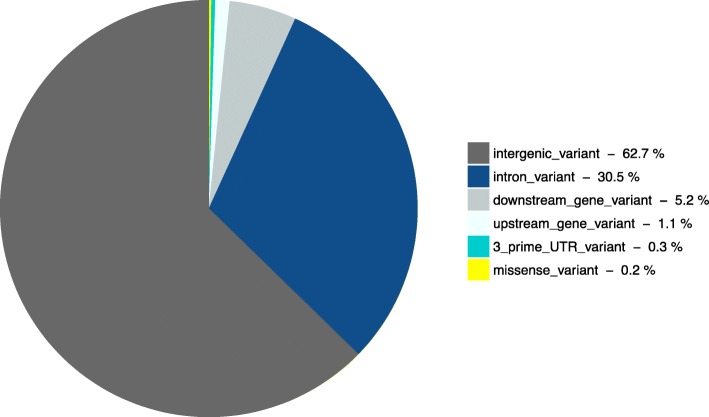
Frequency of VEP consequences terms of the 643 variants of chromosome 5. The upstream and downstream distance to transcript considered were five kilobases

**Fig. 2:**
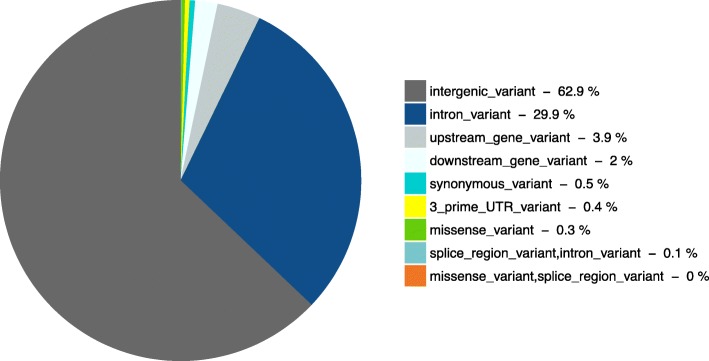
Frequency of VEP consequences terms of the 3732 variants of chromosome 14. The upstream and downstream distance to transcript considered were five kilobases

Based on the VEP analyses, the majority (close to 62% for both QTL regions) of the genomic variants were in intergenic regions which can control genes nearby [39] (Figs. 1 and 2). Otherwise, 0.2% (one locus) and 0.3% (11 loci) were classified as missense variants for BTAs 5 and 14 respectively, being non-synonymous mutations resulting in a codon for different amino acids (Table 4).

**Table 4:** Missense sequence variants concordant with the sires’ QTL status of the chromosomes (BTA) 5 and 14

BTA	Variant ID	Position	Allele	Gene	AA	SIFT
5	rs137779125	70,597,511	A	*MTERF2*	R/C	tol (0.16)
14	rs1116146729	20,718,306	A	–	R/W	del (0.00)
	rs518797951	21,079,097	C	*PRKDC*	E/G	del (0.01)
	rs41718998	21,100,385	C	*PRKDC*	K/R	del (0.01)
	Novel	21,620,454	A	*PPDPFL*	R/Q	tol (0.09)
	rs109800133	23,161,253	C	*RB1CC1*	T/A	tol (1.00)
	Novel	23,612,293	C	*RGS20*	L/P	del (0.00)
	rs461823670	23,996,758	A	*RP1*	G/S	tol (0.47)
	rs450031362	23,997,203	G	*RP1*	D/G	tol (0.11)
	rs137722134	23,998,860	G	*RP1*	I/R	tol (0.07)
	rs439817527	23,998,899	T	*RP1*	T/I	tol (1.00)
	rs109065397	21,104,637	C	*PRKDC*	S/G	tol (0.50)

Position = variant position in base pair; Allele = the variant allele used to calculate the consequence; Gene = gene symbol; AA = reference and variant amino acids; Codons = the alternative codons with the variant base in upper case; SIFT = SIFT prediction score; tol = tolerated; del = deleterious.

The missense variant found for the QTL on BTA 5 is the SNP rs137779125 located at base pair 70,597,511, and is present on the Illumina® BovineHD BeadChip, with MAF of 0.19 in a sample of genotyped sires. The marker is in a coding region of the *MTERF2* (Mitochondrial Transcription Termination Factor 2) gene.

The 11 missense variants identified on the QTL of BTA 14 were located within coding region of the genes ENSBTAG00000040351 (1), *PRKC* (3), *PPDPFL* (1), *RB1CC1* (1), *RGS20* (1) and *RP1* (4). Of these, the markers rs137722134 (23998860 bp – MAF: 0.22) and rs109065397 (21104637 bp) are present on the BovineHD BeadChip and BovineSNP50 BeadChip, respectively.

## Discussion

Weller et al. [40] proposed the use of the daughter design as a method for QTL detection in dairy cattle. In that approach, genotypic information is recorded for sires and their daughters, with phenotypic observations made on the daughters. In this study, a similar approach was used, and a sire was deemed to be heterozygous for the QTL if the difference between its two haplotype alleles was greater than the given threshold (*alpha* < 0.10). Israel et al. [41] similarly reported that sires can be accurately identified as heterozygous for a QTL when the difference between their two haplotype alleles was greater than a given threshold.

The reconstructed haploblocks spanned the previously reported significant genomic windows of each chromosome and trait presented in Oliveira Junior et al. [16]. The number of haplotype alleles identified in a population is sensitive to the width used to define the haploblocks. This width could be reduced with the inclusion of more genotyped heifers in the dataset. Narrower haploblocks are likely to increase the frequency of common haplotype alleles among sires, giving more statistical power to the analysis.

The detection of haplotype alleles associated with the impairment of female fertility can have an important economic impact. Adams et al. [42] reported a loss of approximately $420 million in Holstein dairy cattle due to a mutation in the *APAF1* (apoptotic protease activating factor 1) gene located in the haplotype HH1 on BTA 5 (~ 63 Mb). In addition to HH1, Cole et al. [43] reported the presence of seven others haplotypes related to fertility traits in Holsteins, with a known causative mutation having been reported for three of these (HH3 [44], HH4 [45] and HH5 [46]). Moreover, the knowledge of haplotype regions associated with traits of interest is essential to a better understanding of these traits, identifying genes and biological pathways underlying these QTL.

In the present study, the strategy of regressing the sequence variants present on the SNP chip haplotype alleles in QTL regions of heterozygous sires has been used. The haplotype alleles identified to be segregating for NF were not well represented in any of the sequenced sires, and consequently, no fine map sequence variants were able to be reported for that trait. For HP, the fine mapping the QTL regions identified 641 sequence variants on BTA 5, and 733 sequence variants on BTA 14, that were associated with the trait. Among those, one was classified as missense variants for BTA 5, and eleven had the same classification for BTA 14. These variants were detected in the genes *MTERF2*, *RTBC*, miRNA ENSBTAG00000037306, ENSBTAG00000040351, *PRKDC*, and *RGS20,* which are known to influence reproductive biological processes.

The identification of new genetic variants allows us to better understand the biological complexity of traits of interest. Knowledge of causal mutations should increase accuracy of genomic predictions as well as the genetic progress of the population [8]. According to Brondum et al. [47], a gain of up to 4% in reliability can occur if causative loci are fitted in genomic prediction models. Weller et al. [15] argued that investments in QTN detection are worth it even if these new variants lead to an increase of only 1% in the rate of genetic gain. The identification of new genetic variants is also key information for identifying targets for gene editing.

Imputation procedures are normally used to increase SNP density for fine mapping studies [48, 49]. However, we did not consider conventional imputation approaches due to the small number of available sequenced animals which would limit the accuracy of phasing the sequence alleles. The use of the 1000 Bulls Genomes Project [50] sequence database was not a viable option since most of the animals within that dataset are taurine, whereas the animals in this experiment were indicine. All available animals were used for the prediction of haplotype effects and for the association between sequence variant and the haplotypes allelic with major effects. However, it is important to note that, even considering a conservative threshold for estimation, the small number of sequenced sires could underrepresent the real heterozygosity status of the variant and lead us to have excluded that variant from further consideration.

The over-representation analyses were used to identify classes of genes or proteins that are over-represented in a large set of genes or proteins and that may have an association with the phenotypes. Among the detected MeSH terms associated with HP, “Prolactin” (D011388) is well known to be related to mammary tissue development, immune function, heat tolerance, and reproduction. Polymorphisms of prolactin gene have been associated as mediators of physiological responses of heat stress on cattle, playing a role in the reproductive performance of dairy cows managed in tropical environmental conditions [51, 52]. Leyva-Corona et al. [53] have suggested the use of genomic markers associated with prolactin could help the genetic improvement of fertility traits of cattle raised in warm climates. The “Circadian rhythm” KEGG term was significantly associated with HP, being related to the circadian rhythm, which is an internal biological clock regulating the timing observed in many physiological phenomena, such as sleep and wakefulness, changes in metabolic activity, and cell cycle transition [54, 55]. The gene *CRY1* (cryptochrome circadian regulator 1) is one of the circadian genes which are known to encode transcription-suppressing factors that control the circadian clock in mammals [56]. Of the 643 variants that were detected to significantly affect HP, eight were within intronic regions of the *CRY1* gene (Table 3). Amano et al. [57, 58] concluded that *CRY1* transcripts (such as other circadian genes) are important for the development of oocytes and preimplantation embryos in cattle. Reiter et al. [59] also discussed the importance of circadian rhythms, highlighting the regulation role of daily melatonin secretion. The authors [59] concluded that melatonin plays a key role in oocyte maturation, success ovulation and the development of corpus luteum.

The VEP classification identified a missense variant (G/A) at 70597511 base pair of BTA 5. This mutation is a known SNP (rs137779125), included in Bovine HD Illumina BeadChip, and within a codon region of the *MTERF2* gene. Although this gene is not well characterized, other genes of this family (*MTERF3* and *MTERF4*) have been related to embryonic death and a phenotype that is lethal to mouse embryos [60, 61].

Two novel putative causal variants were detected on BTA 5. The first one was in the upstream region (5’UTR, promoter region) of the *RTBC* gene, which is associated with biological processes such as utero embryonic development and placenta development [62]. The second was in the downstream gene region, where the biotype was annotated as novel miRNA (ENSBTAG00000037306). Such miRNAs are small RNA molecules that function in post-transcriptional regulation of gene expression [63].

There were 11 missense-variants identified in protein coding regions in the haploblock of BTA 14 (Tables 3 and 4). Six of them were classified as deleterious according to the SIFT score and were within the genes ENSBTAG00000040351, *PRKDC*, and *RGS20*. The SIFT approach uses sequence homology to predict whether a substitution affects protein function and consequently the phenotypes [64].

The gene ENSBTAG00000040351 encodes Vomeronasal type-1 receptor protein, which presents the molecular function of G-protein coupled receptor and transducer. G-protein coupled receptors mediates most of the physiological responses to hormones, neurotransmitters and environmental stimulants being directly related to reproductive hormones mainly in females [65]. There is evidence that an orphan G protein-coupled receptor is involved in estrogen signaling in the brain and plays a function as a membrane estrogen receptor [66]. Currently, it is known that G protein-coupled receptors are widely expressed in mammalian tissues [67], suggesting that it may have an important regulatory role in reproductive traits.

Protein kinase, DNA-activated, catalytic polypeptide (*PRKDC*) gene encodes DNA-dependent protein kinase (DNA-PK), which is a nuclear protein serine/threonine kinase that is a molecular sensor of DNA damage. A previous study demonstrated that *PRKDC* expression increases due to embryonic genome activation and acts enzymatically activating the existing proteins in blastocysts [68]. Based on their findings, the authors indicated this gene had a key role on the rate of embryo development, interferon tau expression, and trophoblast development. They also suggested that *PRKDC* is needed during early bovine embryo development.

The family of regulators for G protein signaling (*RGS*) contains regulatory and structural components of G protein-coupled receptor complexes, which mediate several cellular processes. *RGS20* was previously identified as a hub gene in a transcriptional profile study on pre-implantation of bovine embryos developed *in vivo*. *RGS20* have been described as an important regulator of gene expression and stage transition in early embryo development [69].

Haplotype alleles related to HP and NF and also new variants affecting HP have been identified. The KEGG term “Circadian rhythm” was associated with HP, and variants on genes *MTERF2*, *RTBC*, and the miRNA ENSBTAG00000037306 were reported as significantly affecting the trait. There were 11 novel variants identified on BTA 14, with six of them spread on genes ENSBTAG00000040351, *PRKDC*, and *RGS20*. Based on the literature [70], these genes are known to have influence on reproductive biological processes. Sequence data represents a useful resource in biological research, supporting the identification of novel variants and the fine mapping of causative mutations for traits of interest.

## Conclusion

The identification of heterozygous sires for QTL and the use of whole-genome sequencing data allowed for the identification of potential causal mutations and candidate genes associated with reproductive traits in a Nellore population. Genomic variants on genes *MTERF2*, *RTBC*, miRNA ENSBTAG00000037306, ENSBTAG00000040351, *PRKDC*, and *RGS20,* which are known to have influence on reproductive biological processes, were detected. Given the limitations of the datasets used for the present study, further studies considering expression analyses and other “omics” approaches could support an improved ability to infer the causality of sequence variants identified. Nevertheless, these findings could contribute to a better understanding of the genetic control and biological processes involved in female fertility and could lead to innovative DNA-based selection strategies.

## Supplementary information

**Additional file 1: Figure S1.** Posterior distribution of the differences in estimated values of heifer pregnancy between two haplotype alleles on chromosome 5 in those six Nellore sires where the contrast was significant (posterior *Alpha* < 0.10).

**Additional file 2: Figure S2.** Posterior distribution of the differences in estimated values of heifer pregnancy between two haplotype alleles of the chromosome 14 in those six Nellore sires where the contrast was significant (posterior *Alpha* < 0.10).

**Additional file 3: Figure S3.** Posterior distribution of the differences in estimated values of heifer pregnancy between two haplotype alleles on chromosome 18 in five Nellore sires where the contrast was significant (posterior *Alpha* < 0.10).

**Additional file 4: Figure S4.** Posterior distribution of the differences in estimated values for number of antral follicles between two haplotype alleles one chromosome 8 in six Nellore sires where the contrast was significant (posterior *Alpha* < 0.10).

**Additional file 5: Figure S5.** Posterior distribution of the differences in estimated values for number of antral follicles between two haplotype alleles on chromosome 11 in fourteen Nellore sires where the contrast was significant (posterior *Alpha* < 0.10).

**Additional file 6: Figure S6.** Posterior distribution of the differences in estimated values for number of antral follicles between two haplotype alleles on chromosome 22 in five Nellore sires where the contrast was significant (posterior *Alpha* < 0.10).

**Additional file 7: Table S1.** Significant (*P*-value < 0.05) MeSH terms related to genes located in the QTL of chromosome 14.

## Data Availability

The datasets used and/or analyzed during the current study are available from the corresponding author on reasonable request.

## Abbreviations

bp: base pair
BTA: *Bos taurus* autosome
GATK: Genome analysis toolkit
GGP: GeneSeek® genomic profiler
Haploblock: Haplotype block
HD: High density
HH: Holstein haplotype
HP: Heifer pregnancy
KEGG: Kyoto encyclopedia of genes and genomes
MAF: Minor allele frequency
Mb: Megabase
MeSH: Medical subject headings
NF: Number of antral follicles
PCR: Polymerase chain reaction
QTL: Quantitative trait loci
QTN: Quantitative trait nucleotide
SNP: Single nucleotide polymorphism
VEP: Variant effect predictor
WGS: Whole-genome sequencing
