# Supplementary figures and images for: Fine mapping of genomic regions associated with female fertility in Nellore beef cattle based on sequence variants from segregating sires
Source: J Anim Sci Biotechnol. 2019 Dec 16;10:97. doi: 10.1186/s40104-019-0403-0 (PMC6913038; doi:10.1186/s40104-019-0403-0)

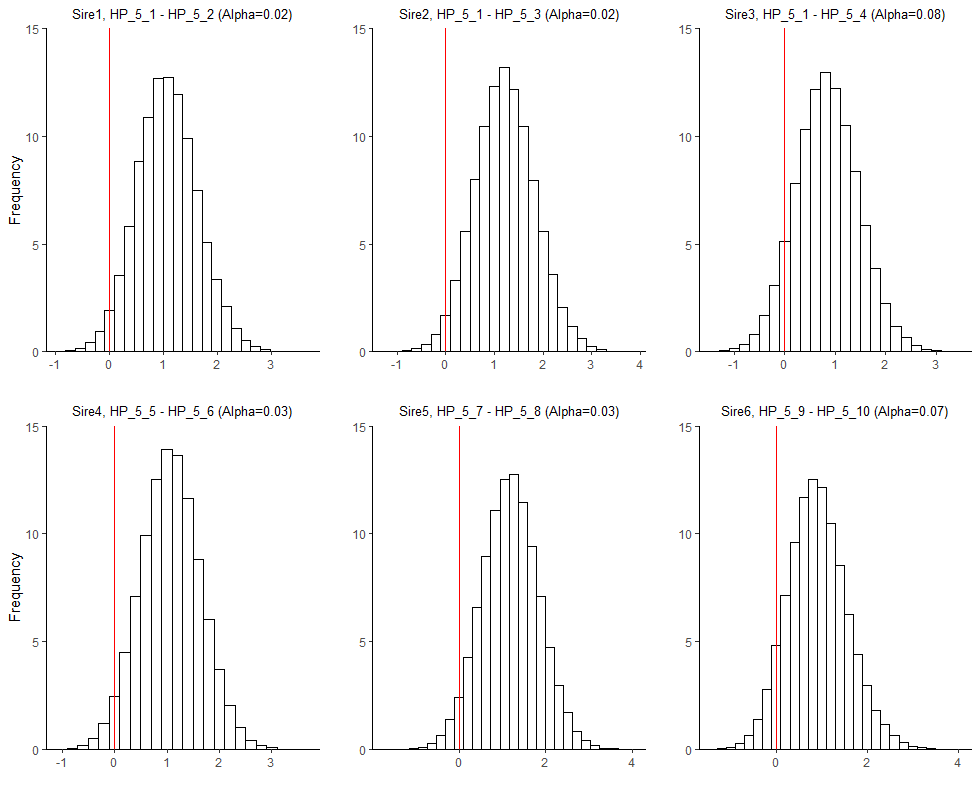

Supplement: Supplementary file 1 — Additional file 1: Figure S1. Posterior distribution of the differences in estimated values of heifer pregnancy between two haplotype alleles on chromosome 5 in those six Nellore sires where the contrast was significant (posterior Alpha < 0.10). [file 40104_2019_403_MOESM1_ESM.docx]

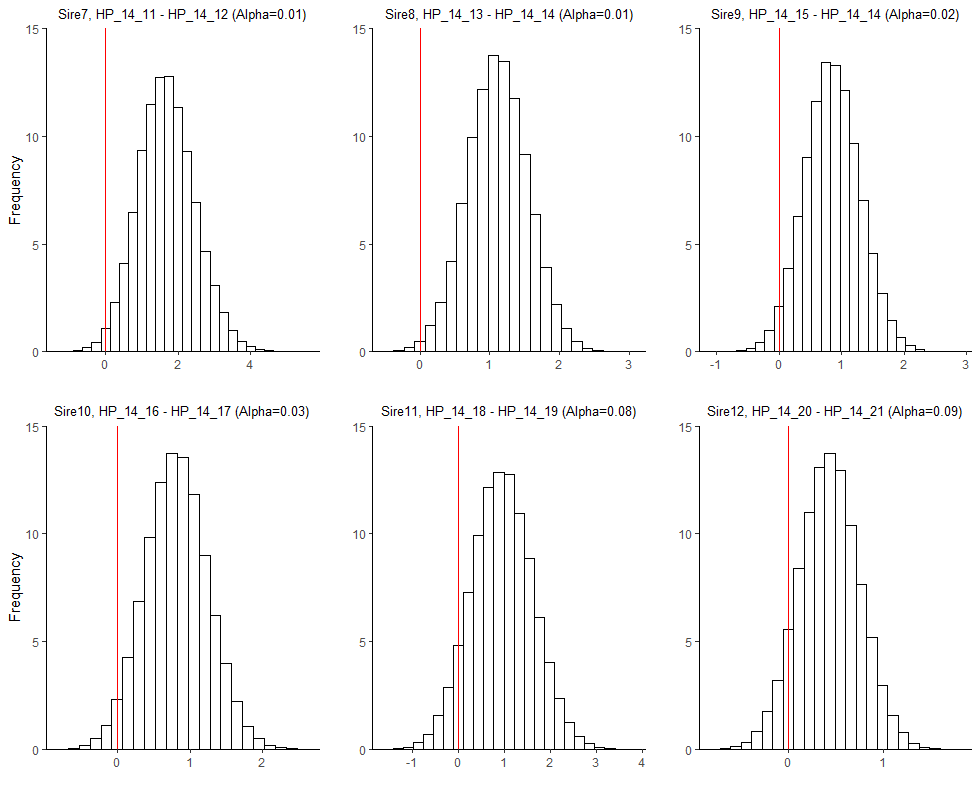

Supplement: Supplementary file 2 — Additional file 2: Figure S2. Posterior distribution of the differences in estimated values of heifer pregnancy between two haplotype alleles of the chromosome 14 in those six Nellore sires where the contrast was significant (posterior Alpha < 0.10). [file 40104_2019_403_MOESM2_ESM.docx]

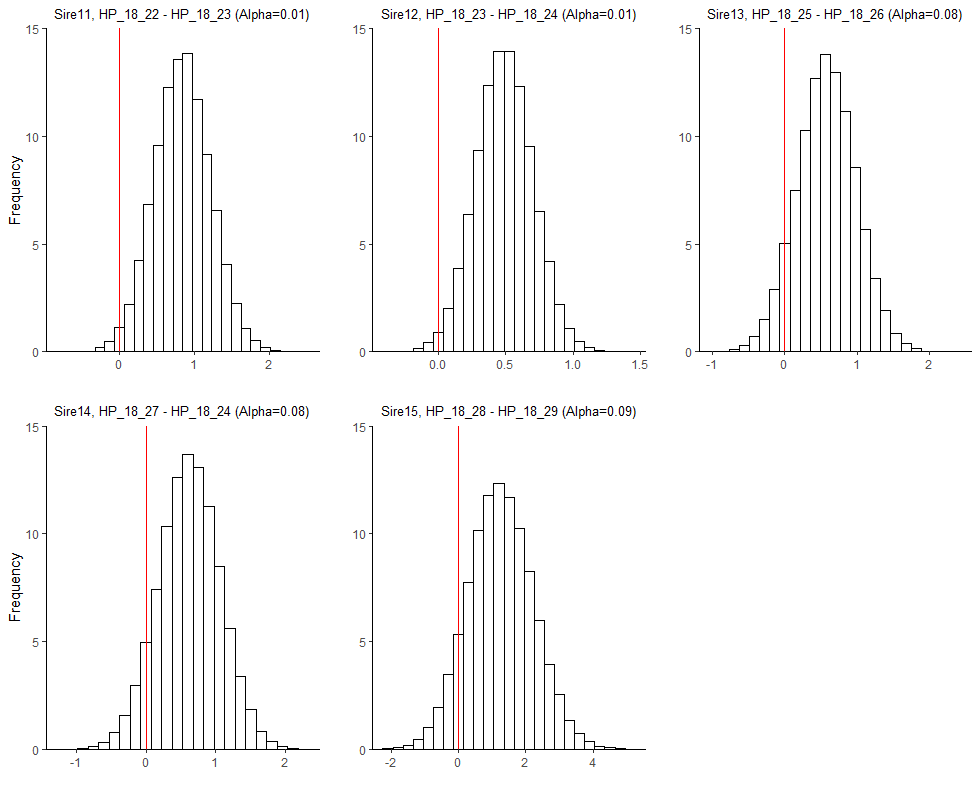

Supplement: Supplementary file 3 — Additional file 3: Figure S3. Posterior distribution of the differences in estimated values of heifer pregnancy between two haplotype alleles on chromosome 18 in five Nellore sires where the contrast was significant (posterior Alpha < 0.10). [file 40104_2019_403_MOESM3_ESM.docx]

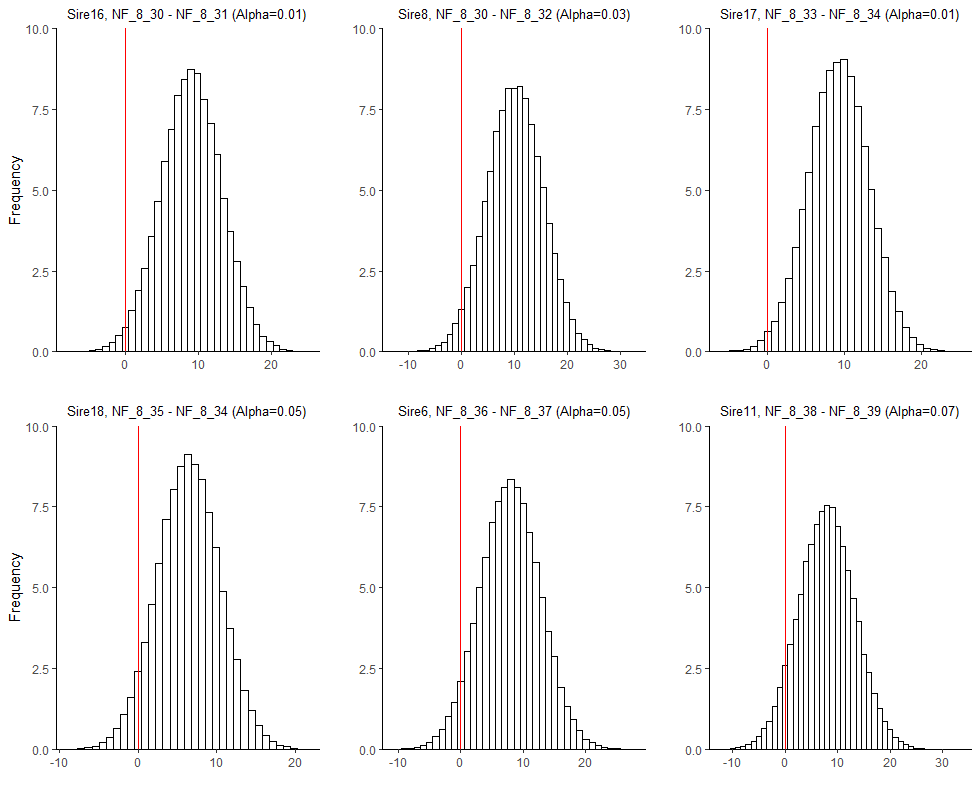

Supplement: Supplementary file 4 — Additional file 4: Figure S4. Posterior distribution of the differences in estimated values for number of antral follicles between two haplotype alleles one chromosome 8 in six Nellore sires where the contrast was significant (posterior Alpha < 0.10). [file 40104_2019_403_MOESM4_ESM.docx]

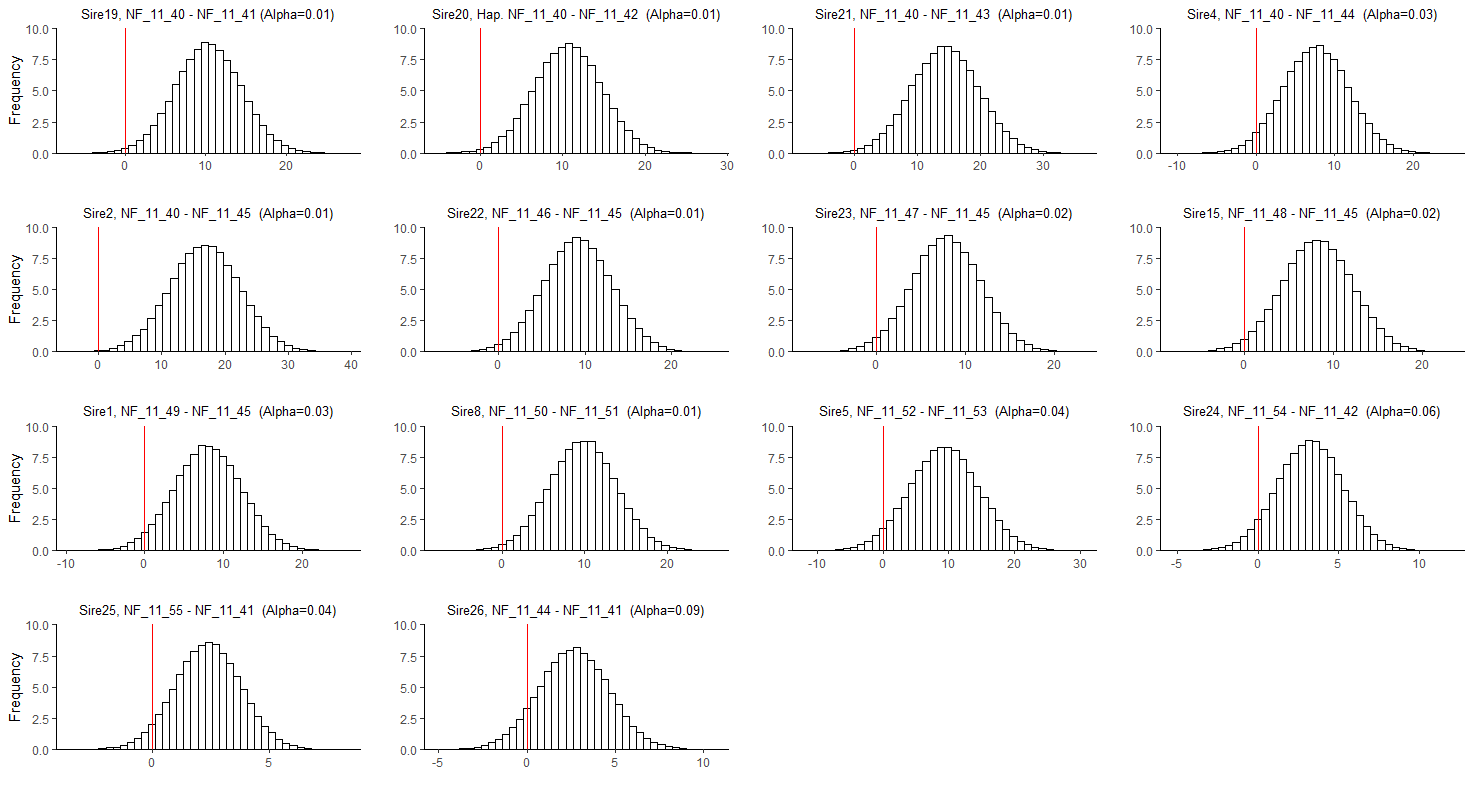

Supplement: Supplementary file 5 — Additional file 5: Figure S5. Posterior distribution of the differences in estimated values for number of antral follicles between two haplotype alleles on chromosome 11 in fourteen Nellore sires where the contrast was significant (posterior Alpha < 0.10). [file 40104_2019_403_MOESM5_ESM.docx]

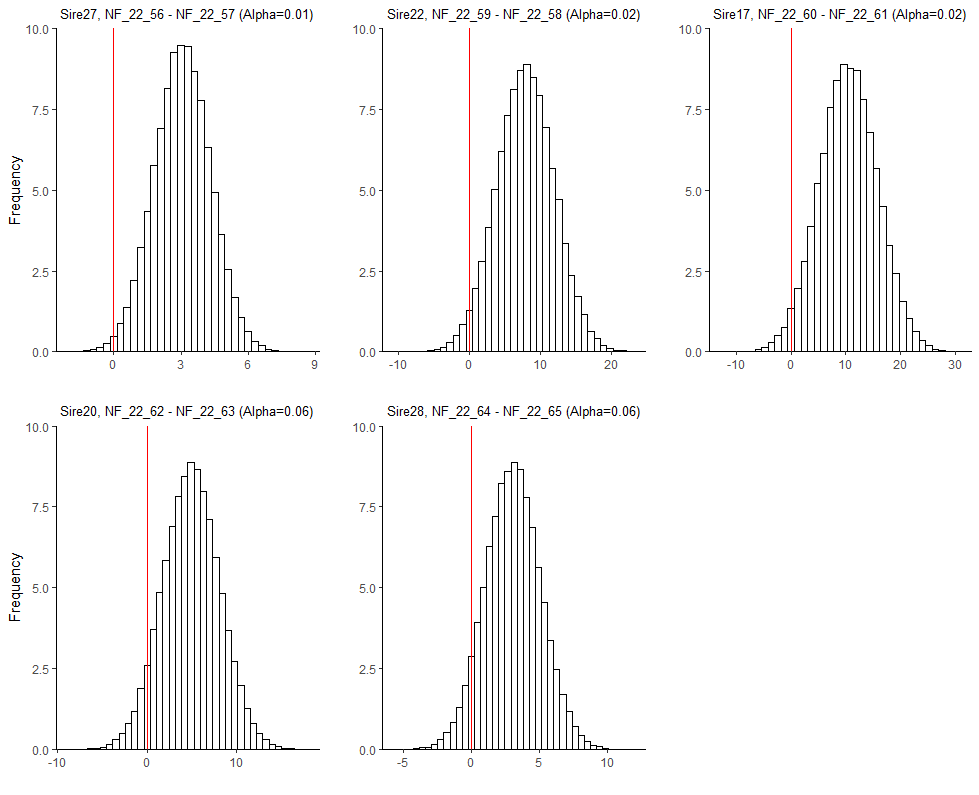

Supplement: Supplementary file 6 — Additional file 6: Figure S6. Posterior distribution of the differences in estimated values for number of antral follicles between two haplotype alleles on chromosome 22 in five Nellore sires where the contrast was significant (posterior Alpha < 0.10). [file 40104_2019_403_MOESM6_ESM.docx]
