# Supplementary material for: Fine mapping of genomic regions associated with female fertility in Nellore beef cattle based on sequence variants from segregating sires
Source: J Anim Sci Biotechnol. 2019 Dec 16;10:97. doi: 10.1186/s40104-019-0403-0 (PMC6913038; doi:10.1186/s40104-019-0403-0)
Supplement: Supplementary file 7 — Additional file 7: Table S1. Significant (P-value < 0.05) MeSH terms related to genes located in the QTL of chromosome 14. [file 40104_2019_403_MOESM7_ESM.docx]

| Mesh term ID | Mesh term name | #genes | #sig. genes | P-value |
| --- | --- | --- | --- | --- |
| D007626 | Kell Blood-Group System | 1 | 1 | 1.3E-03 |
| D033983 | Adaptor Protein Complex mu Subunits | 1 | 1 | 1.3E-03 |
| D010171 | Palmitoyl Coenzyme A | 3 | 1 | 3.9E-03 |
| D051401 | Aquaporin 4 | 3 | 1 | 3.9E-03 |
| D017473 | Receptors, Opioid, kappa | 4 | 1 | 5.1E-03 |
| D011957 | Receptors, Opioid | 5 | 1 | 6.4E-03 |
| D044385 | GTP-Binding Protein alpha Subunits | 5 | 1 | 6.4E-03 |
| D033962 | Adaptor Protein Complex 2 | 6 | 1 | 7.7E-03 |
| D048057 | Mitogen-Activated Protein Kinase 10 | 6 | 1 | 7.7E-03 |
| D018747 | RNA, Chloroplast | 8 | 1 | 1.0E-02 |
| D018013 | Receptors, Neuropeptide | 9 | 1 | 1.2E-02 |
| D025262 | Vacuolar Proton-Translocating ATPases | 10 | 1 | 1.3E-02 |
| D009419 | Nerve Tissue Proteins | 192 | 2 | 1.8E-02 |
| D020710 | RGS Proteins | 14 | 1 | 1.9E-02 |
| D012343 | RNA, Transfer | 15 | 1 | 1.9E-02 |
| D019308 | Palmitic Acid | 15 | 1 | 2.2E-02 |
| D018388 | Codon, Terminator | 17 | 1 | 2.3E-02 |
| D020962 | Heterotrimeric GTP-Binding Proteins | 18 | 1 | 2.5E-02 |
| D015293 | Transducin | 20 | 1 | 2.5E-02 |
| D017494 | Proton Pumps | 20 | 1 | 2.5E-02 |
| D020690 | GTPase-Activating Proteins | 20 | 1 | 2.5E-02 |
| D012367 | RNA, Viral | 21 | 1 | 2.7E-02 |
| D004229 | Dithiothreitol | 24 | 1 | 3.0E-02 |
| D016244 | Guanosine 5'-O-(3-Thiotriphosphate) | 25 | 1 | 3.2E-02 |
| D000900 | Anti-Bacterial Agents | 31 | 1 | 3.9E-02 |
| D006180 | Proton-Translocating ATPases | 32 | 1 | 4.0E-02 |
| D033942 | Adaptor Proteins, Vesicular Transport | 32 | 1 | 4.0E-02 |
| D011388 | Prolactin | 34 | 1 | 4.3E-02 |
| D026901 | Membrane Transport Proteins | 35 | 1 | 4.4E-02 |
| D008081 | Liposomes | 37 | 1 | 4.7E-02 |
| D020558 | GTP Phosphohydrolases | 37 | 1 | 4.7E-02 |
| D006160 | Guanosine Triphosphate | 38 | 1 | 4.8E-02 |

#genes = number of genes; #sig. genes = number of significant genes.
